# Pancreatic panniculitis in a patient with pancreatic-type acinar cell carcinoma of the liver – case report and review of literature

**DOI:** 10.1186/s12885-016-2184-6

**Published:** 2016-02-20

**Authors:** Sebastian Zundler, Ramona Erber, Abbas Agaimy, Arndt Hartmann, Franklin Kiesewetter, Deike Strobel, Markus F. Neurath, Dane Wildner

**Affiliations:** Department of Medicine 1, University Hospital Erlangen, Ulmenweg 18, 91054 Erlangen, Germany; Institute of Pathology, University Hospital Erlangen, Erlangen, Germany; Department of Dermatology, University Hospital Erlangen, Erlangen, Germany

**Keywords:** Pancreatic panniculitis, Acinar cell carcinoma, Pancreatitis, Paraneoplastic, Lipase, Liver

## Abstract

**Background:**

Pancreatic panniculitis is a rare condition, which has only been described in relation with pancreatic diseases up to now. It is characterized by necrotizing subcutaneous inflammation and is thought to be triggered by adipocyte necrosis due to systemic release of pancreatic enzymes with consecutive infiltration of neutrophils. We present the first case of a patient with pancreatic panniculitis caused by pancreatic-type primary acinar cell carcinoma (ACC) of the liver and without underlying pancreatic disease.

**Case presentation:**

A 73-year old Caucasian female patient was referred to our department with painful cutaneous nodules persisting for eight weeks and with marked lipasemia (~15000 U/l; normal range <60 U/l). Four weeks prior, several liver lesions had been detected. Empiric treatment with steroids did not show any effect. A biopsy of the skin nodules revealed “pancreatic” panniculitis, while abdominal imaging with ultrasound, computed tomography and magnetic resonance imaging detected no abnormal pancreatic findings. Ultrasound-guided biopsy of the liver lesions showed infiltrates of an ACC. The patient died soon thereafter. Autopsy failed to reveal any other primary for the ACC, so that a pancreatic-type ACC of the liver was diagnosed by exclusion.

One hundred thirty cases of pancreatic panniculitis published within the last 20 years are reviewed. ACC of the pancreas is the most common underlying neoplastic condition. Patients with associated neoplasm are significantly older, take longer to be diagnosed and have higher lipase levels than patients with underlying pancreatitis.

Extrapancreatic pancreatic-type ACC is very rare, but shows the same biological features as ACC of the pancreas. It is believed to develop from metaplastic or ectopic pancreatic tissue. Up to now, no pancreatic panniculitis in extrapancreatic ACC has been described.

**Conclusion:**

Pancreatic panniculitis should always be included in the differential diagnosis of lipolytic panniculitic lesions. It can be regarded as a facultative paraneoplastic phenomenon.

When suspected, a thorough work-up for identification of the underlying disease is mandatory and extrapancreatic lesions (e.g. liver) should also be considered. While administration of octreotide or steroids can sometimes alleviate symptoms, immediate treatment of the associated condition is the only effective management option.

## Background

Chiari was the first to describe the development of panniculitic lesions in patients with pancreatitis in 1883 [1]. Since then, several case reports and small case series have reported focal or generalized panniculitis in association with pancreatic diseases like acute or chronic pancreatitis, pancreatic carcinoma (ductal adenocarcinoma, acinar cell carcinoma, neuroendocrine carcinoma) or intraductal papillary mucinous neoplasm (IPMN) [2–6].

Up to 45 % of patients with pancreatic panniculitis show subcutaneous panniculitic nodules before the causal disease is recognized [2]. Therefore, these nodules can serve as an early and valuable clue to diagnosis of the underlying condition and trigger measurement of serum pancreatic enzymes, abdominal imaging or biopsy procedures. Histologic evaluation of the cutaneous lesions will typically reveal lobular neutrophilic necrotizing panniculitis intermingled with specific necrotic anucleate adipocytes called “ghost cells” [7].

The mechanism underlying the formation of panniculitic nodules in pancreatic panniculitis is poorly understood. However, it is commonly believed that systemically released pancreatic enzymes such as lipase and amylase cause distant lipolysis and fat necrosis with consecutive inflammatory reaction [8]. This is supported by the finding that the necrotic tissue stains positive for lipase [9]. However, serum levels of pancreatic enzymes do not correlate with clinical findings and similarly, in vitro experiments suggest that this explanation is not sufficient [10].

In addition to the cutaneous manifestation, arthritis is often found in patients with pancreatic panniculitis, clinically referred to as pancreatitis panniculitis polyarthritis (PPP) syndrome. It is thought that pancreatic enzymes are also able to trigger necrosis and inflammation in the synovium [11]. Furthermore, there are reports about panniculitis in the bone marrow, at submucosal sites or within the thoracic or peritoneal cavity [2, 11, 12].

Acinar cell carcinoma (ACC) is a rare pancreatic malignancy, representing about 1 % of all primary pancreatic neoplasms [13]. ACC is the most common malignancy found in patients with pancreatic panniculitis [14] and symptoms of pancreatic panniculitis can be found in up to 16 % of ACC patients [4]. On very rare occasions, pancreatic-type ACC can also arise as a primary neoplasm at extrapancreatic locations, such as liver, stomach, jejunum and colon [15–18]. In such cases, extrapancreatic ACC is believed to originate from either ectopic, metaplastic of transdifferentiated pancreatic tissue and shares biologic features with primary pancreatic ACC [15].

Here, we report the first case of pancreatic panniculitis in association with a primary pancreatic-type ACC of the liver without underlying pancreatic disease. Moreover, we present a review of case reports and case series of pancreatic panniculitis from the last 20 years, summarizing important knowledge and data about this disease entity.

## Case presentation

**Fig. 1:**
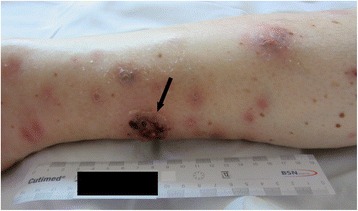
Several panniculitic lesions on the right leg of the patient, one of them (→) shortly after having spontaneously drained brownish-oily fluid

A 73-year-old Caucasian female patient was referred to our department for further work-up of painful cutaneous lesions (Fig. 1) and several masses within her liver.

Eight weeks prior, she had observed an erythematous nodule on her right chest. Subsequently, similar cutaneous lesions had developed on her arms and legs, and later also on her buttocks and back. She did not report any abdominal complaints. Outpatient treatment with topical and systemic steroids based on a suspicion of erythema nodosum (EN) did not yield substantial effect.

**Fig. 2:**
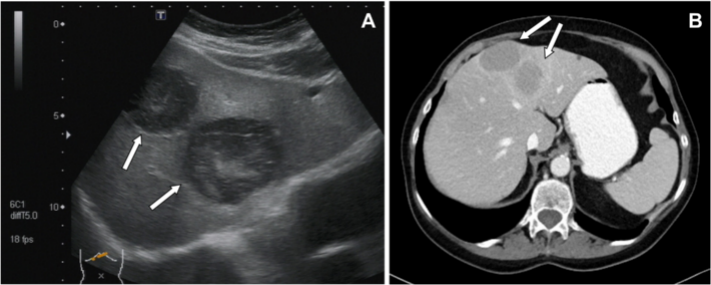
Imaging of the liver lesions (→) with ultrasound (**a**) and CT (**b**)

Four weeks prior, several liver lesions had been detected by ultrasound and were interpreted as metastases of a previously treated breast cancer. Additional imaging with computed tomography (CT) and magnetic resonance imaging (MRI) had been carried out (Fig. 2) and confirmed the liver lesions.

**Fig. 3:**
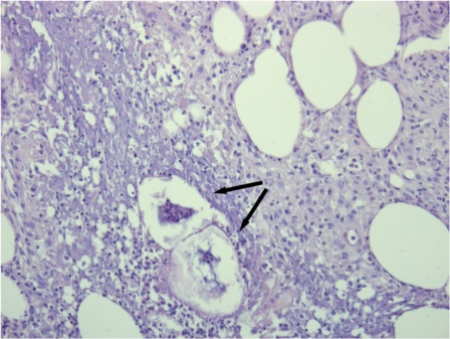
Biopsy from a skin lesion showing lobular neutrophilic, necrotizing panniculitis and so called “ghost cells” (→)

**Fig. 4:**
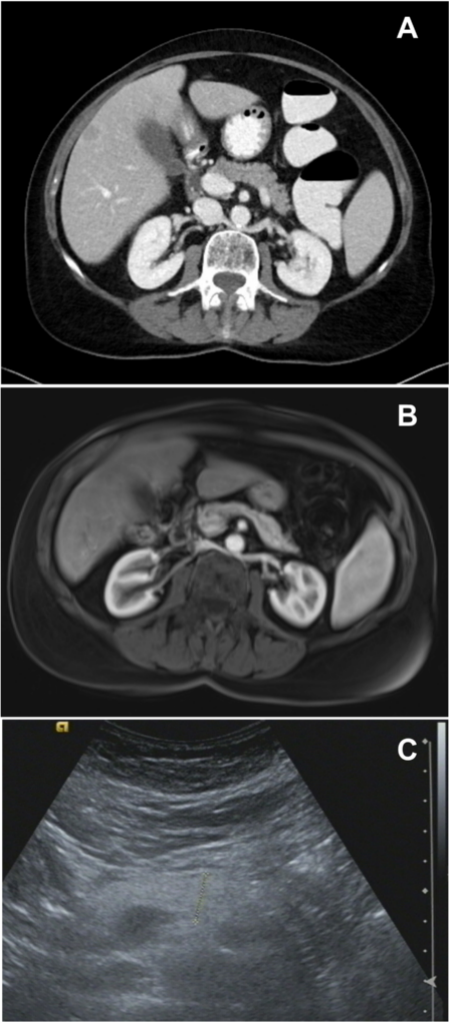
Abdominal imaging showing no evidence of pancreatic pathology. **a** CT. **b** MRI. **c** ultrasound

As the nodules on her skin continued to spread and became increasingly painful, she was presented to the Department of Dermatology in our clinic. There, another attempt of steroids and an intensified local therapy resulted in no improvement of her clinical condition. Due to raising inflammatory parameters a work-up for possible infectious causes and an antibiotic therapy with piperacillin/tazobactam, and later with meropenem were initiated. A colonoscopy revealed two small polyps, which were completely removed. Pancreatic enzymes were markedly elevated. A punch biopsy of one of the skin lesions was obtained showing a lobular necrotizing panniculitis with “ghost cells” compatible with pancreatic panniculitis (Fig. 3). CT, MRI and repeated ultrasound examinations (Fig. 4) did not reveal any pathological findings in the pancreas. In contrast enhanced CT multiple sharply-bounded liver lesions were visualized in both liver lobes. Compared with the CT obtained during outpatient care, the lesions had progressed in size and measured from 1 cm to 6 cm. The perfusion pattern was non-hypervascular and the density was hypointense, partly comparable with the density of water. No necrotic areas were described within the lesions.

Because of a progressive worsening of her clinical condition and increasing laboratory markers of inflammation, the patient was referred to our Department of Internal Medicine. She complained about intensive pain all over her skin and required increasing dose rates of opioid analgetics. She did not report any weight loss, night sweats, fever, nausea or vomiting, abdominal pain or problems with food intake. Her past medical history was remarkable for invasive ductal breast cancer diagnosed in 1982 with local recurrences in 1990 and 2008. Moreover, a superficial spreading malignant melanoma had been treated in 2011 and a coronary artery disease with percutaneous coronary intervention in 2008 was reported. Family history was significant for malignant melanomas in all siblings and her mother. Continuous medication included acetyl salicylic acid, lercanidipine, metoprolol, enalapril and pravastatin with no recent change. No allergic condition was known.

On examination she was in poor general condition (ECOG performance status 4), tachycardic (102 bpm), slightly tachypnoeic (22/min) and normotensive (128/78 mmHg). Her temperature was 36.9 °C. Subcutaneous erythematous and painful nodules of 2–5 cm size were noticed throughout her integument. Some of them were spontaneously draining a brownish oily fluid. Moreover, more than 200 melanocytic nevi were observed on her skin. Examination of the head, especially focusing on the salivary glands was unremarkable. There was no pain on abdominal palpation, the liver was palpable 2 cm under the right costal arch and bowel sounds were normal. There was a positive tap sign on both patellae.

Laboratory results of interest were: leukocyte count 21.5 * 10^3/μl (ref. 4–10 * 10^3/μl), hemoglobin 10.0 g/dl (ref. 12–16 g/dl), ASAT 52 U/l (ref. < 35U/l), GGT 235 U/l (ref. <40 U/l), AP 186 U/l (ref. 35–105 U/l), lipase 14747 U/l (ref. < 60 U/l) and CRP 237 mg/l (ref. < 5 mg/l). Alpha-Amylase, uric acid, ACE, CEA, CA19-9 and AFP were within normal range. Serology for Yersinia enterocolitica and pseudotuberculosis was negative, as well as testing for Mycobacterium tuberculosis and atypical mycobacteria. Rheumatologic testing including ANAs and ANCAs was unremarkable.

Screening for possible infectious foci did not reveal any other source explaining the elevated CRP. Therefore, it was attributed to the skin lesions, which displayed clinical signs of inflammation and were partly draining pus in the further course. However, as microbiological evaluation was not able to prove any causative organism and inflammation markers were not substantially declining despite escalation of antibiotic treatment with additional vancomycin, skin lesions were classified as sterile. Leukocytosis was explained by concomitant steroid therapy.

Ultrasound displayed several liver lesions in both lobes with a maximum size of 53 mm. The pancreas was homogeneous and free of focal lesions. The pancreatic duct was not dilated and no avascular areas could be detected upon administration of ultrasound contrast agent. Ultrasound-guided puncture of one of the liver masses was performed leading to the histopathological diagnosis of a pancreatic-type ACC.

Unfortunately, the condition of the patient had severely deteriorated in the meantime with further exacerbation of pain, increasing tachycardia and hypotension. Therefore, no tumor-specific treatment could be initiated. The patient died ten days after admission to our ward.

### Pathological and autopsy findings

**Fig. 5:**
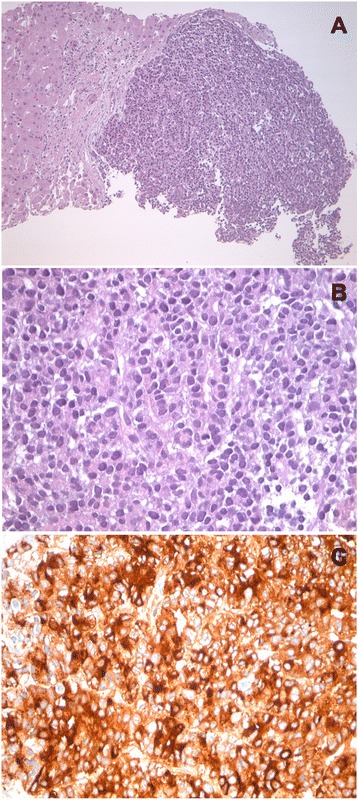
Histomorphology and immunohistochemistry of the liver tumor (from core biopsy). **a** core biopsy of the liver showing liver tissue adjacent to the acinar cell carcinoma, haematoxylin/eosin staining, 10-fold magnification. **b** compact acinar structures and trabeculae seen at higher magnification, haematoxylin/eosin staining, 40-fold magnification. **c** The tumor cells stained strongly for trypsin, 40-fold magnification

Histopathological analysis of the core biopsy obtained from the liver mass revealed a cellular epithelial neoplasm composed of monomorphic polygonal or rounded cells arranged in compact acinar and trabecular structures (Fig. 5a, b). Immunohistochemical study revealed strong expression of pancytokeratin (KL-1) with variable expression of CK7 and diffuse strong cytoplasmic expression of trypsin (Fig. 5c), but lipase and amylase were negative. All other markers in the differential diagnosis were negative (CK5, CK20, HepPar-1, Synaptophysin, Chromogranin A, NSE, CD56, TTF1, ER, PR, protein S100, GATA3 and PAX8). These findings including in particular the strong and specific expression of trypsin confirmed the diagnosis of pancreatic-type ACC in the liver.

Autopsy confirmed several liver masses measuring up to six centimeters in size. There was no evidence of a salivary gland tumor or a primary pancreatic tumor. Additionally, review of the slides from the patient’s previous breast cancer confirmed a breast cancer of no special type and excluded the possibility of acinar-like differentiation. Thus, the previous breast cancer was also unrelated to the patient's ACC. Cause of her death was attributed to multiorgan failure due to severe systemic inflammatory response syndrome.

Final diagnosis was pancreatic panniculitis due to primary pancreatic-type acinar cell carcinoma of the liver.

Taking into account the conspicuous accumulation of malignancies in our patient and her family, genetic analysis for familial atypical multiple mole-melanoma (FAMMM) syndrome was recommended to her relatives.

### Review of literature

In addition to the presented case, 130 reports on pancreatic panniculitis were identified in the English literature between January 1994 and November 2014 by using the search terms “pancreatic panniculitis”, “subcutaneous fat necrosis AND pancreas” and “lipase hypersecretion syndrome” in PubMed and by checking results for appropriate cross-references.

Including the above case, all 131 cases (Table 1) were analyzed in respect to available data on age and gender of the patients, the underlying condition, additional symptoms, the sequence of the appearance of panniculitis and the diagnosis of the underlying disease, laboratory values and the outcome. The stated percentages refer to the respective number of cases including data on the analyzed parameter. Statistical analysis was performed with IBM SPSS Statistics (IBM, Armonk, NY, USA) using Student’s T-test or Fisher’s exact test where applicable. *p* < 0.05 was considered significant. Graphs were generated with SigmaPlot (Systat, San Jose, CA, USA).

**Table 1:** Overview of pancreatic panniculitides described in English literature between January 1994 and November 2014

Patient	Ref.	Age	Sex	Underlying condition	Outcome of skin lesions/follow-up
1	our pat.	73	f	ACC of the liver	death ten weeks after first skin lesion
2	[53]	55	m	metastatic pancreatic NEC	n.r.
3	[54]	38	m	acute pancreatitis	resolution
4	[7]	63	m	acute pancreatitis	resolution
5	[55]	81	f	hemosuccus pancreaticus	death 19 weeks after first skin lesion
6	[56]	26	f	acute pancreatitis	resolution
7	[57]	27	f	acute pancreatitis	resolution
8	[58]	34	m	chronic pancreatitis	n.r.
9	[59]	63	f	acute pancreatitis	resolution
10	[60]	83	m	pancreatic ACC	resolution of skin lesions after surgery, death 34 months after first skin lesion
11	[60]	75	m	Acute pancreatitis	resolution
12	[60]	60	m	Large pancreatic neoplasm	n.r.
13	[61]	68	f	metastatic pancreatic ACC	death six months after first skin lesion
14	[62]	61	f	acute pancreatitis	resolution
15	[63]	63	f	acute pancreatitis	resolution
16	[64]	49	m	acute pancreatitis	resolution
17	[47]	69	m	hepatic metastasis of previous-ly resected pancreatic ACC	resolution after metastasectomy, follow-up n.r.
18	[65]	71	f	chronic pancreatitis with pseudocyst	n.r.
19	[66]	66	f	serous cystadenoma of the pancreas	n.r.
20	[67]	39	m	chronic pancreatitis	death six weeks after first skin lesion
21	[68]	20	f	pseudopapillary pancreatic tumor, chronic pancreatitis	death eleven weeks after first skin lesion
22	[8]	38	f	acute pancreatitis	resolution
23	[69]	56	f	acute pancreatitis	death within a few days
24	[70]	79	m	pancreatic ACC	n.r.
25	[71]	17	f	acute pancreatitis	resolution
26	[72]	54	m	pancreatic ACC	Response to octreotide, regression of skin lesions after resection, follow-up two months (i.e. eleven months after first skin lesion)
27	[73]	81	f	pancreatic tumor with hepatic metastases	n.r.
28	[74]	64	m	anastomotic leakage after Whipple procedure	death 50 days after first skin lesion
29	[75]	79	f	pancreatic ACC	regression after resection, follow-up 14 months after resection (i.e. 16 months after first skin lesion)
30	[35]	60	f	pancreatic adenocarcinoma	n.r.
31	[35]	58	m	malignant neoplasia of the tail of the pancreas	n.r.
32	[76]	44	f	pancreas transplant rejection	resolution
33	[77]	63	f	metastatic pancreatic adenocarcinoma	n.r.
34	[78]	38	m	pancreatic pseudocyst-inferior vena cava fistula	resolution after Roux-en-Y pseudocyst-jejunostomy
35	[40]	82	m	pancreatic ACC	death 2.5 months after first skin lesion
36	[46]	79	m	pancreatic ACC	death two months after first skin lesion
37	[79]	2,5	m	acute pancreatitis with pseudocyst	n.r.
38	[80]	45	m	chronic pancreatitis	resolution after placement of endoprothesis
39	[81]	18	w	acute pancreatitis	resolution
40	[82]	10	w	acute pancreatitis with pseudocyst	resolution after endosonographic cysto-gastrostomy
41	[83]	69	m	gastric adenocarcinoma with pancreas metastasis	death 14 weeks after first skin lesion
42	[84]	58	m	pancreatic pseudocyst-portal vein fistula	death five days after admission
43	[85]	84	m	pancreatic tumor	death two months after first skin lesion
44	[86]	40	m	chronic pancreatitis	n.r.
45	[87]	84	f	acute pancreatitis, liver lesions ten years after resection of colonic carcinoma	initially resolution, death three months later
46	[2]	65	f	acute pancreatitis	death from gangrenous cholecystitis
47	[2]	64	f	acute pancreatitis	resolution
48	[2]	70	f	pancreatic carcinoma	death
49	[2]	58	m	pancreatic carcinoma	death
50	[2]	21	f	pancreatic carcinoma	death
51	[2]	75	m	acute pancreatitis	death
52	[2]	75	f	acute pancreatitis	resolution
53	[2]	44	m	chronic pancreatitis	resolution
54	[2]	63	m	pancreatic carcinoma	death
55	[2]	72	m	pancreatic carcinoma	death
56	[2]	60	m	pancreatic carcinoma	n.r.
57	[88]	35	m	chronic pancreatitis	n.r.
58	[89]	7	m	acute pancreatitis	resolution
59	[14]	61	m	metastatic NEC of unknown primary site	death a few weeks after first skin lesion
60	[42]	50	f	metastatic ACC	regression of skin lesions and tumor under octreotide, gemcitabine, streptozocin and doxorubicin; return after discontinuation; response to restart of therapy; follow-up 9 months after first skin lesion
61	[90]	74	m	pancreatic ACC	death 3.75 months after first skin lesion
62	[91]	61	m	metastatic ACC	n.r.
63	[92]	79	m	metastatic pancreatic NEC	Regression under cefazolin, dexamethasone and NSAID; death 13 months after first skin lesion
64	[93]	50	m	acute pancreatitis	death 38 days after first skin lesion
65	[94]	4	m	acute pancreatitis	resolution
66	[11]	45	m	acute pancreatitis	Regression under NSAID and prednisone
67	[95]	72	m	acute pancreatitis	resolution
68	[96]	52	f	pancreatic carcinoma	death six months after first skin lesion
69	[97]	29	m	acute pancreatitis	resolution
70	[23]	75	f	hepatic metastases of adeno-carcinoma of unknown origin	death 15 weeks after first skin lesion
71	[6]	78	m	metastatic pancreatic NEC	death two months after first skin lesion
72	[6]	75	m	pancreatic adenocarcinoma	regression under irradiation, follow-up n.r.
73	[98]	67	f	IPMN	partial pancreatectomy, follow-up n.r.
74	[99]	51	m	chronic pancreatitis	regression of skin lesions under conservative treatment
75	[100]	49	f	pancreas transplant rejection	resolution
76	[101]	89	f	acute pancreatitis	resolution
77	[42]	59	m	hepatic metastases of pre-viously resected pancreatic ACC	death several weeks after first skin lesion
78	[102]	67	m	metastatic pancreatic adenocarcinoma	regression under irinotecan, cisplatin, mito-mycin; death twelve months after first skin lesion
79	[103]	62	f	IPMN	resection, follow-up n.r.
80	[22]	60	m	metastatic pancreatic acinar cell cystadenocarcinoma	death seven weeks after admission
81	[104]	13	m	chronic pancreatitis with pseudocyst	regression after cystogastrostomy
82	[41]	72	m	pancreatic NEC	regression after resection, no reappearance with liver metastases, death 15 months after first skin lesion
83	[25]	58	m	HCC	death four months after first skin lesion
84	[105]	88	m	metastatic pancreatic NEC	Death eight weeks after first skin lesion
85	[106]	42	f	acute pancreatitis	resolution
86	[107]	63	m	chronic pancreatitis	resolution after distal pancreatectomy and pancreatic duct dilation
87	[108]	34	m	acute pancreatitis	resolution
88	[109]	21	f	acute pancreatitis	death from retroperitoneal hemorrhage
89	[110]	61	f	metastatic pancreatic ACC	death one year after first skin lesion
90	[3]	70	f	IPMN	resolution after resection
91	[3]	53	f	IPMN	resolution after resection
92	[111]	37	f	acute pancreatitis with pseudocyst	resolution after surgery
93	[111]	50	m	acute pancreatitis with pseudocyst	resolution after stone extraction from the pancreatic duct
94	[112]	71	m	pancreatic adenocarcinoma	n.r.
95	[113]	60	f	pancreatic ACC	regression after distal pancreatectomy, follow-up 28 months after first skin lesion
96	[113]	54	m	pancreatic ACC	death six weeks after first skin lesion
97	[114]	56	m	chronic pancreatitis	n.r.
98	[115]	53	m	actue pancreatitis	resolution
99	[48]	67	m	metastatic pancreatic ACC	Regression after TACE of four liver metasta-ses, death 14 weeks after first skin lesion
100	[116]	n.r.	n.r.	chronic pancreatitis	resolution after placement of pancreatic duct stent
101	[117]	31	m	acute pancreatitis	resolution
102	[118]	57	f	acute pancreatitis	resolution
103	[119]	60	f	pancreatic ACC	resolution after resection, follow-up n.r.
104	[120]	41	m	acute pancreatitis	death 22 days after admission
105	[44]	79	f	metastatic pancreatic ACC	Death 20 weeks after first skin lesion
106	[121]	45	m	chronic pancreatitis	resolution
107	[122]	59	f	acute pancreatitis	resolution
108	[123]	81	m	pancreatic ACC	n.r.
109	[124]	69	m	pancreatic ACC	n.r.
110	[125]	15	f	acute pancreatitis	death nine days after first skin lesion
111	[126]	49	m	acute pancreatitis	regression under antibiotic treatment, then slight progression, follow-up seven weeks
112	[127]	66	m	pancreatic ACC	resolution after resection, follow-up n.r.
113	[128]	67	m	chronic pancreatitis	death two months after first skin lesion
114	[12]	69	m	pancreatic ACC	resolution after distal pancreatectomy and adjuvant radio-chemotherapy, follow-up 6 months
115	[129]	29	f	acute pancreatitis	resolution
116	[130]	29	m	pancreatic pseudocyst-portal vein fistula	no new lesions after surgery
117	[131]	75	m	pancreatic tumor	Death several months after first skin lesion
118	[131]	39	m	chronic pancreatitis with pseudocyst	n.r.
119	[132]	62	m	acute pancreatitis	resolution
120	[133]	77	f	pancreatic tumor	death five months after first skin lesion
121	[134]	54	f	chronic pancreatitis	resolution after ESWL and endoscopic dilation of the pancreatic duct
122	[135]	47	f	acute pancreatitis	resolution
123	[136]	7	m	acute pancreatitis	n.r.
124	[137]	69	m	chronic pancreatitis with pseudocyst	resolution
125	[138]	33	f	acute pancreatitis	n.r.
126	[139]	46	f	chronic pancreatitis	death four months after first skin lesion
127	[140]	55	m	chronic pancreatitis	n.r.
128	[5]	62	m	acute pancreatitis	resolution
129	[141]	61	f	metastatic ACC	resolution after surgery, follow-up n.r.
130	[142]	80	m	pancreatic ACC	resolution after surgery, death from metastatic disease after 18 months
131	[143]	n.r.	m	pancreatic ACC	n.r.

Overall, 65 cases (49.6 %) were due to acute or chronic pancreatitis and 60 cases (45.8 %) had an underlying neoplastic condition. In six cases (4.6 %) other reasons were present, e.g. pancreas transplant rejection or pancreaticovascular fistula (Table 2).

**Table 2:** Etiology of pancreatic panniculitis

Pancreatitis		Neoplastic conditions		Other	
Acute	34.4 %	pancreatic ACC	19.8 %	fistula	2.3 %
Chronic	15.3 %	n. r.	9.9 %	transplant rejection	1.5 %
		NEC	4.6 %		
		adeno-carcinoma	3.8 %	anastomotic leakage	0.8 %
		IPMN	3.1 %		
		other	4.6 %		
Total	49.6 %	total	45.8 %	total	4.6 %

While near half of the cases are caused by acute or chronic pancreatitis, another 45.8 % are associated with neoplastic conditions (other: acinar cystadenocarcinoma, ACC of the liver, serous cystadenoma, HCC, gastric carcinoma with pancreatic infiltration, adenocarcinoma of unknown primary)

**Fig. 6:**
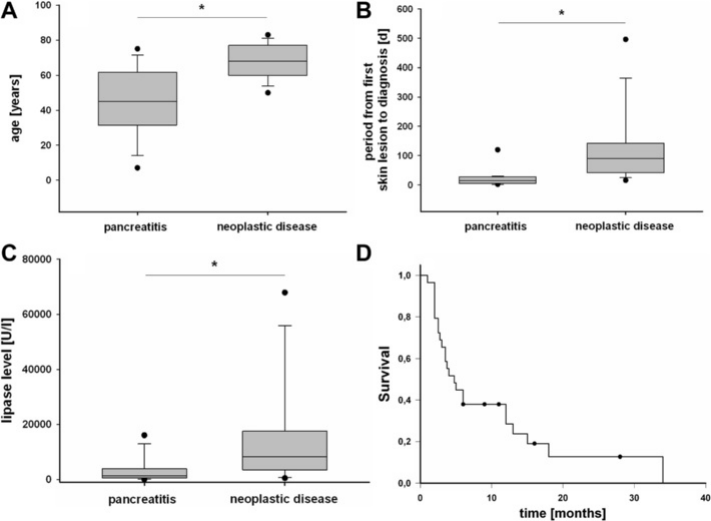
Comparison of patients with pancreatitis and neoplasm underlying pancreatic panniculitis (**a**-**c**): **a** Patients with neoplastic conditions are significantly older than patients with pancreatitis (66.0 +/− 13.0 years vs. 44.7 +/− 20.5 years, *p* < 0.001). **b** Underlying malignancy is diagnosed significantly later than underlying pancreatitis (134 +/− 135 days vs. 20 +/− 26 days, *p* < 0.001). **c** Tumor patients have significantly higher lipase levels than pancreatitis patients (16611 +/− 20772 vs. 5324 +/− 14436 U/l, *p* < 0.01). **d** Kaplan-Meier plot of survival after appearance of the first panniculitis lesion in patients with pancreatic panniculitis associated with malignancy. Median survival is 4.75 months (*n* = 29)

Patients with pancreatic panniculitis had a mean age of 54.8 years. Yet, patients with neoplastic causes were significantly older than individuals with pancreatitis (Fig. 6a). 57.4 % of the patients were male with no difference in sex distribution between underlying pancreatitis and malignancy.

In 48.9 %, cutaneous lesions were noted prior to the diagnosis of the underlying disease. The mean duration from appearance of the first lesion to diagnosis was 85 days +/− 110 days (range: 2–540 days; median 42 days). This period was significantly longer when pancreatic panniculitis was due to a neoplasm than when a pancreatitis was present (Fig. 6b). Moreover, the portion of patients developing panniculitis before the diagnosis of the underlying condition was by trend higher in patients with neoplastic disease (66.7 %) than in patients with pancreatitis (48.3 %; *p* = 0.06).

A PPP syndrome with additional signs of arthritis was present in 49 cases (37.4 %).

One hundred twelve case reports (85.5 %) contained information on the serum levels of at least one pancreatic enzyme. In all but two of these reports (1.8 %) either amylase or lipase were elevated – in one of these two cases only amylase had been measured. The mean level of lipase was 11560 U/l +/− 19010 U/l (range 7–89700 U/l, median 3942.5 U/l). Again, patients with pancreatitis and neoplastic conditions differed markedly with tumor patients having significantly higher lipase levels (Fig. 6c). ROC analysis identified a lipase level of 4414 U/l as best cut-off value with higher values having a sensitivity of 73.0 % and a specificity of 82.1 % for the diagnosis of a neoplastic cause (AUC = 0.785, 95 % CI 0.68 to 0.89).

Only limited data was available concerning survival and follow-up. 12 patients with pancreatitis (21.4 %) died from complications. For underlying malignancy, follow-up data was available for 29 patients. A Kaplan-Meier plot of survival was computed, yielding a median survival of 4.75 months after appearance of the first skin lesion (Fig. 6d).

### Discussion

Panniculitis is a clinical finding, which can be caused by various etiologic factors including infectious, immunologic and neoplastic conditions [19–21].

In our case, numerous causes could be excluded, while others were very unlikely: No infectious organism could be detected directly or indirectly. Continuous medication was unchanged and unsuspicious for causing erythema nodosum. Imaging had not yielded any evidence of malignancy other than the finally diagnosed ACC. Rheumatologic disease was judged unlikely based on consultation with a rheumatologist.

Therefore, regarding laboratory data and histologic results pancreatic panniculitis was the only possible diagnosis.

Our case of pancreatic panniculitis is noteworthy for two reasons: The absence of pancreatic disease and the extrapancreatic manifestation of pancreatic-type ACC. The combination of both has not been previously described in the literature. Pancreatic panniculitis without definite proof of pancreatic disease is found in four cases in the literature: Beltraminelli et al. [22] report a case of acinar cell cystadenocarcinoma of presumably pancreatic origin metastatic to the liver. However, clear evidence of a pancreatic primary tumor was absent on imaging. Freireich-Astmann et al. [23] describe the history of a patient with hepatic metastases of an adenocarcinoma of unknown primary. CT did not show any pancreatic lesion and immunohistochemistry was negative for CA19-9 and CK19. Aznar-Oroval et al. present a case of gastric adenocarcinoma with hepatic metastases in association with pancreatic panniculitis, but without clinical or radiologic findings of pancreatic disease [24]. And finally, Corazza et al. [25] report about a patient with multifocal hepatocellular carcinoma (HCC) and missing pancreatic lesions in CT.

However, in all cases, no autopsy for definite verification of the absence of pancreatic disease was performed. Amylase or lipase were elevated in each of the cases, but could not be explained by clinical, radiologic or histological findings in all but Beltraminelli et al.’s case. While existence of a primary hepatic acinar cell cystadenocarcinoma should have been discussed in this case, findings are inconclusive in the other three.

The HCC described by Corazza showed “trabecular structures and acinar aspects”, features suggestive of or consistent with ACC [15]. As immunohistochemistry is not reported, the possibility of a pancreatic-type ACC of the liver cannot be fully excluded in that case.

Primary extrapancreatic ACC is extremely rare and only six cases of ACC originating in the liver have been described to date [15, 26, 27]. Diagnosis of pancreatic-type ACC originating from the liver requires exclusion not only of an occult pancreatic primary, but also of primaries at other possible sites, such as breast [28] or salivary glands [29]. In our case, neither clinical nor radiological evidence for another primary was present, which was finally verified by autopsy findings. Moreover, re-analysis of the samples of the previously treated breast cancer excluded a hitherto undiscovered acinar cell carcinoma of the breast.

Because of the rarity of primary ACC of the liver, no typical pattern can be specified in the different imaging modalities up to now. So far, most of the cases described were initially misclassified as one of the most common primary liver malignancies, such as HCC or cholangiocellular carcinoma (CCC), due to their imaging appearance. Moreover, a recent study on imaging findings in pancreatic ACC also reported a high variability in several parameters analyzed [30]. Thus, a thorough histological work-up of specimens after a resection or core biopsy is required to ensure the correct diagnosis [15, 26, 27].

What could be objected to the diagnosis of an ACC of the liver in our case is the multifocality of the liver lesions, which is suggestive for metastatic disease. However, despite thorough work-up no other primary was found. Furthermore, it is worth noting that ACC is normally relatively large in size by the time of diagnosis [4], which makes an occult primary rather unlikely. In addition, multifocal growth of primary liver tumors is not unusual, e.g. in intrahepatic CCC [31, 32] and HCC [33, 34]. Indeed, primary hepatic ACC might originate from acinar trans-differentiation of biliary progenitor cells, thus representing the acinar counterpart of hepatic cholangiocarcinoma [15].

In an analysis of more than 130 cases of pancreatic panniculitis described in the last 20 years, we could show that nearly half of the cases are associated with an internal malignancy. Current concepts of the pathogenesis of pancreatic panniculitis suggest a role of pancreatic enzymes produced or released by these tumors [8, 9]. Therefore – though only rarely so named [35] – pancreatic panniculitis should be regarded as facultative paraneoplastic condition [36] and a tumor screening, especially for pancreatic tumors, should always be included in the diagnostic work-up.

The analysis of different parameters of these cases revealed significant differences between patients with pancreatic panniculitis and associated neoplasm or pancreatitis. On average, patients with a tumor are older and have higher lipase levels. Moreover, it takes longer until a diagnosis is made in these cases. A lipase cut-off value of 4414 U/l is able to differentiate between underlying pancreatitis and neoplasm with a sensitivity and specificity comparable with CA 19–9 in ductal adenocarcinoma vs. benign pancreatic disease [37].

Regarding the epidemiology and the natural course of malignancy and pancreatitis these results are not very surprising. However, these items can provide a first orientation, which etiology has to be primarily suspected. Like this, they may trigger a particularly intensive search for tumors in older patients with high lipase levels and a long-lasting history of panniculitis.

This is even more important as pancreatic panniculitis seems to be a hallmark of poor prognosis in tumor patients. Median survival in the cases with underlying malignancy and included follow-up data was 4.75 months after appearance of the first skin lesion.

Of course, this retrospective analysis has significant limitations as it is exclusively based on case reports. Though, it is the first systematic evaluation of survival in pancreatic panniculitis and poor outcome is remarkable, because over 50 % of the included cases were ACC patients, which otherwise have considerably better survival [38, 39].

Due to the rarity of the disease, clear therapeutic algorithms for ACC are missing. Since most of the cases present with distant metastases only a subset of patients qualifies for resection [39]. Therefore, cancer therapy is often limited to palliative approaches like chemotherapy or ablative treatment. As in our case, patients often suffer heavily from the pain caused by their skin lesions and analgetic therapy is frequently not sufficiently able to reduce pain [12, 14, 22, 40, 41]. Thus, palliative treatment strategies are very important for symptom control as well.

Octreotide has been reported to alleviate symptoms in some cases [42–44]. Chemotherapeutic agents reported to be used in patients with pancreatic panniculitis and underlying ACC include gemcitabine and the FOLFIRI regime [45, 46]. Furthermore, one case with resolution of panniculitis following metastasectomy [47] and one case with marked symptom reduction after transarterial chemoembolization (TACE) of hepatic metastases [48] are described in literature. Some success in the treatment of pancreatic ACC has been reported with the use of FOLFOX [49], FOLFIRINOX [50], cisplatin/etoposide [51] and gemcitabine in various combinations including erlotinib [52].

## Conclusion

To our best knowledge, this is the first report of pancreatic panniculitis in a patient with primary ACC of the liver.

The possibility of pancreatic panniculitis should always be included in diagnostic considerations regarding panniculitic lesions. Therefore, a cutaneous biopsy should be obtained, pancreatic enzymes should be measured and abdominal imaging should be performed as early as possible. When diagnosed, pancreatic panniculitis has to be regarded as a facultative paraneoplastic syndrome and appropriate tumor screening or biopsy procedures have to be undertaken. This is especially important in older patients with high lipase levels and long-lasting symptoms. Regarding the presented case, tumors of extrapancreatic primary must also be considered.

Pancreatic-type ACC is the malignancy most often associated with pancreatic panniculitis. It can not only originate from the pancreas but also from the liver, which can be diagnosed, when other primary sites have been excluded.

Pancreatic panniculitis in association with malignancy seems to be linked with poor prognosis. Thus, early diagnosis is necessary to improve survival and ease symptoms, e.g. by resection or chemotherapy. Symptomatic therapy with octreotide seems worth trying. Further studies are required to define standard therapeutic strategies for unresectable ACC.

## Consent

During her lifetime, the patient consented orally to the use of her patient history and all the related images and information for scientific purposes. After the patient’s death her daughter gave written consent for the publication of the case.

## Abbreviations

ACC: acinar cell carcinoma
ACE: angiotensin-converting enzyme
AFP: alpha fetoprotein
ANA: anti-nuclear antibody
ANCA: anti-neutrophil cytoplasmatic antibody
AP: alkaline phosphatase
AST: aspartate transaminase
CA 19–9: carbohydrate antigen 19–9
CCC: cholangiocellular carcinoma
CEA: carcinoembryonic antigen
CK: cytokeratin
CRP: c-reactive protein
CT: computed tomography
ECOG: Eastern cooperative oncology group
EN: erythema nodosum
ESWL: extracorporal shock wave lithotripsy
f: female
FOLFIRI: folinic acid, 5-fluoruracil, irinotecan
FOLFIRINOX: folinic acid, 5-fluoruracil, irinotecan, oxaliplatin
FOLFOX: folinic acid, 5-fluoruracil, oxaliplatin
GGT: gamma glutamyl transferase
HCC: hepatocellular carcinoma
IPMN: intraductal papillar mucinous neoplasm
m: male
MRI: magnetic resonance imaging
n.r.: not reported
NEC: neuroendocrine carcinoma
NSAID: non-steroidal anti-inflammatory drugs
PPP syndrome: pancreatitis panniculitis polyarthritis syndrome
TACE: transarterial chemoembolization
